# Thyroglobulin as a Sensitive Biomarker of Iodine Status in Mildly and Moderately Iodine-Deficient Pregnant Women

**DOI:** 10.1007/s12011-023-03986-5

**Published:** 2023-12-04

**Authors:** Yujie Jiang, Simeng Gu, Zhe Mo, Xueqing Li, Chenyang Liu, Yahui Li, Guangming Mao, Yuanyang Wang, Fanjia Guo, Xiaoming Lou, Xiaofeng Wang

**Affiliations:** 1 Department of Environmental Health, Zhejiang Provincial Center for Disease Control and Prevention, Hangzhou, 310051 China; 2 Health Science Center, Ningbo University, Ningbo, 315211 China

**Keywords:** Iodine, Thyroglobulin, Pregnancy, Biomarkers

## Abstract

Pregnant women are more susceptible to iodine deficiency. However, there are limitations in existing indicators for the evaluation of iodine nutrition in pregnant women. The study aimed to explore whether thyroglobulin (Tg) can be used as a more sensitive biomarker for pregnant women with mild and moderate iodine deficiency. A repeated-measure study was conducted among 1332 pregnant women in Zhejiang Province, China. Serum and urine specimens were collected at a mean of 10, 17, and 32 weeks of pregnancy, respectively; thyroid-stimulating hormone (TSH), Tg, and urinary iodine concentrations were measured. Linear mixed effects models were used to determine the associations between interaction of iodine concentrations and increasing gestation week and TSH and Tg, where participants were divided by urinary iodine concentration (UIC). The median Tg concentration was 11.56, 11.45, and 12.43 μg/L in the first, second, and third trimesters, respectively. After controlling the covariates, the interaction effects between the iodine status and gestation week were significant for both TSH and Tg (*p* = 0.038 and *p* = 0.007, respectively). TSH increased with the week of gestation in both iodine concentration groups. Tg increased with advancing pregnancy in the iodine-deficient group whereas it did not in the iodine-sufficient group. There was no significant variation in TSH at each trimester, and Tg was higher in the iodine-deficient group than in the iodine-sufficient group. Tg may be a more sensitive iodine status biomarker than TSH for pregnant women with mild-to-moderate iodine insufficiency.

## Introduction

Iodine requirements increased due to physiological changes during pregnancy [1]. It is widely recognized that inadequate iodine intake during pregnancy can result in neurodevelopmental defects in the offspring [2]. It would be less challenging to monitor public health and study the health effects of inadequate iodine intake given that iodine status at individual level was accurately evaluated. However, authorized indicators for the assessment of individual iodine nutrition have not been identified.

Currently, given that more than 90% of the dietary iodine intake is metabolized throughout the urine and the convenience and low cost of urinary iodine measurement, urinary iodine concentration (UIC) from a single-spot urine specimen is the most commonly used index to assess the iodine nutritional status at population level [3]. However, urinary iodine is easily influenced by fluid intake, circadian rhythms, and postprandial peaks and does not provide reliable information about individual iodine status and thyroid health [4]. Although thyroid-stimulating hormone (TSH) is a sensitive biomarker of thyroid dysfunction in newborns [5], the value remains the normal reference range in children and adults with mild iodine deficiency, owing to strict homeostasis regulation [6]. Triiodothyronine (T3) and thyroxine (T4) are the main biologically active thyroid hormones, which directly reflect the thyroid function [7]. However, in the case of mild-to-moderate iodine deficiency, the concentration usually fluctuated within the normal reference range and does not reflect the actual iodine nutritional status of the body [8]. Thyroid volume (TV) is a useful indicator for assessing iodine nutrition in severe iodine deficiency, but it is not a timely indicator in mild-to-moderate iodine deficiency [9]. All above-mentioned indicators have their characteristics, but these indicators do not reflect thyroid function caused by suboptimal iodine intake.

Thyroglobulin (Tg), the most abundant intrathyroidal protein, is synthesized only in the thyroid and reflects long-term iodine intake [6, 10]. When there was an iodine deficiency, significant concentrations of Tg are discharged into the blood circulation; Tg was 18 μg/L when iodine excretion < 50 μg/day; then, Tg concentration decreased with increasing iodine excretion [3, 11]. There was a positive association between serum Tg concentration and thyroid volume [11, 12]. In school-aged children, there was a U-shaped correlation between serum Tg and iodine status [13, 14]. In studies among adults, the concentrations of Tg in the mild deficient iodine region (7.5 μg/L) were substantially higher than those in sufficient iodine region (5.9 μg/L) [12, 15]. A study with repeated data sampling in the same individuals for 1 year demonstrated that serum Tg was added to assess iodine nutrition in subgroups when UIC was used to assess the overall iodine nutrition [16]. These findings suggested that Tg could be used as a biomarker for iodine status in both children and adults.

There is mounting evidence that Tg can be used as a biomarker of iodine nutritional status in adults and children [17]. Studies on animals have also shown an association between urinary iodine and serum Tg [18]. While it has also been reported that serum Tg is negatively correlated with urinary iodine in pregnant women [19], it was also true that there was a non-linear association between urinary iodine and Tg, with greater Tg concentrations found in the groups who had inadequate and excessive iodine intakes [20]. The study of Bath et al. repeated measurements of urinary iodine-to-creatinine ratio, serum TSH, and Tg at 12, 20, and 35 weeks of gestation in 230 Caucasian pregnant women; the results shown that Tg was a sensitive biomarker of iodine deficiency in pregnant women [21]. Although Bath et al.’s study used repeated-measurement approach, the sample size was modest, and the study population was from one hospital.

Therefore, the aim of this study was to explore the validity of maternal serum Tg as a biomarker of iodine status in pregnancy with suboptimal iodine intake based on a repeated-measure, multicenter study design with large samples.

## Materials and Methods

### Study Population

**Fig. 1:**
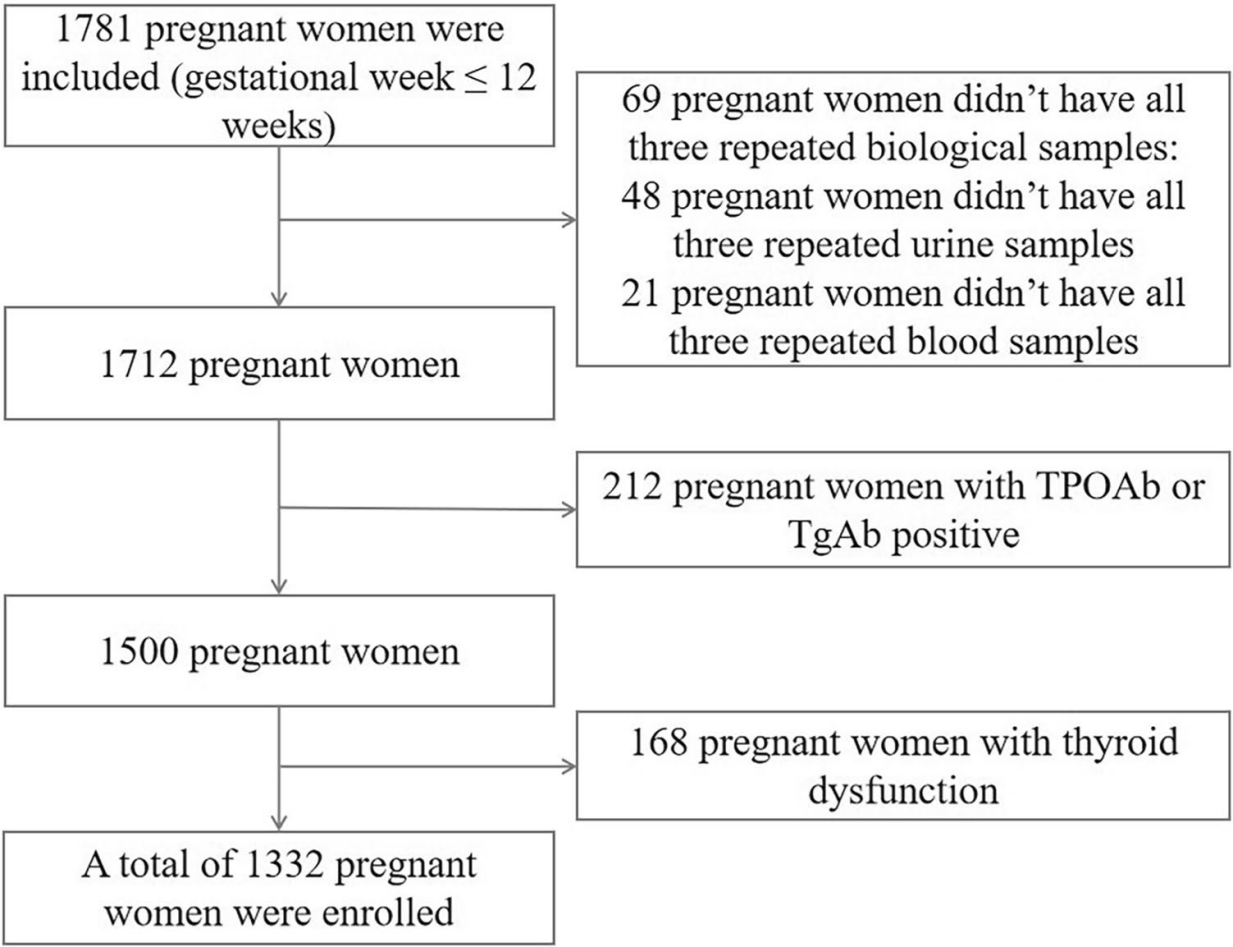
Study flowchart illustrating the study design

 According to the guidelines to assess the iodine deficiency disorder (IDD) national program recommended by the World Health Organization (WHO) [22] and the national IDD surveillance protocol [23], between July 2019 and December 2020, a repeated measure study was conducted among pregnant women in Zhejiang Province, China. Multistage cluster random sampling was performed in this study. First, seven counties were randomly chosen from 90 counties of Zhejiang Province. Second, for each selected county, one Maternal and Child Health Hospital was randomly selected. Finally, for each selected Maternal and Child Health Hospital, 200 pregnant women were recruited randomly during their first hospital visit. Recruitment criteria included women aged 18–45 years who had lived in the county for at least 1 year and plan to live in the county for the next 3 years, those women whose gestational age ≤ 12 weeks, and those who were pregnant with a singleton pregnancy. Excluded were those pregnant women who gestational age > 12 weeks; patients with a history of thyroid disease or any other chronic diseases, and participants who have had an iodine-containing contrast agent injection within 1 year. Antibodies to Tg can interfere with Tg measurements and TSH can be affected by autoimmune thyroid disease [24, 25]. Therefore, for a more accurate analysis concerning how iodine concentrations correlate with thyroid function, those pregnant women who are Tg antibody (TgAb) positive or thyroid peroxidase antibody (TPOAb) positive and individuals with thyroid dysfunction were excluded from the analysis. Finally, the remaining 1332 pregnant women were enrolled. For each participant, they were required to complete a questionnaire at their first hospital visit, and then, blood and urine samples were collected at a mean of 10, 17, and 32 weeks of gestation. Figure 1 shows a flowchart of the process for excluding study participants.

This study was approved by the Ethics Committee of Zhejiang Provincial Center for Disease Control and Prevention and was in accordance with the declaration of Helsinki (Ethics approval number: 2020–047-01). All participants received a signed informed permission following a thorough explanation of the study protocol.

### Laboratory Analyses

#### Serum Thyroid Function Measurements

Fasting venous blood was collected from each participant at a mean of 10, 17, and 32 weeks of gestation. Serum samples were used to assess Tg, TSH, free triiodothyronine (FT3), free thyroxine (FT4), TPOAb, and TgAb using the electrochemiluminescence immunoassay (ECLIA) with a Cobas e411 analyzer (Roche Diagnostics, Germany), along with the compatible calibration materials, reagents, and quality controls. In complying with the recommendations of the manufacturer, quality control was carried out before, during, and after testing. All specimens were examined after quality control samples were tested to ascertain the reliability of the results. The results of the quality control were within the standard range of quality control samples. The coefficient of variation for TSH at 1.5 mIU/L was 3.0%; at the level of 21.5 µg/L, it was 5.0% for Tg; at the level of 5.78 pmol/L, it was 4.0% for FT3; at the level of 16.1 pmol/L, it was 2.0% for FT4; at the level of 88.4 IU/mL, it was 6.0% for TgAb; at the level of 51.2 IU/mL, it was 5.0% for TPOAb. A small number of the measured serum Tg values were under the assay limit of detection (LOD) of 0.04 μg/L; the values below the LOD were replaced with one-half of LOD (0.02 μg/L), which was a common method for handling below-detection values [26]. According to the manufacturer’s cut-offs, the positive TPOAb and TgAb were both > 34 IU/mL and 115 IU/mL, respectively. According to the guideline on diagnosis and management of thyroid diseases during pregnancy and postpartum (2nd edition) issued by the Chinese Society of Endocrinology and the Chinese Society of Perinatology [27], thyroid dysfunction was diagnosed. Thyroid dysfunction was defined as overt hypothyroidism, subclinical hypothyroidism, overt hyperthyroidism, subclinical hyperthyroidism, and isolated hypothyroxinemia.

#### Urinary Iodine Measurements

Spot urine samples from each participant for examination of urinary iodine were taken at around 10, 17, and 32 weeks of gestation. Based on the Sandell-Kolthoff reaction, UIC was detected by the WHO recommended As^3+^-Ce^4+^ catalytic spectrophotometry method with ammonium persulfate digestion. The China National Iodine Deficiency Disorders Reference Laboratory provided the internal quality control samples for urine iodine. Iodine concentrations of all samples were examined in the National Reference Laboratories at the county level. The China National Iodine Deficiency Disorders Reference Laboratory provided the internal quality control samples for urine iodine. The results of the quality control were within the standard range of quality control samples. The coefficient of variation for UIC was 2.0% at 79.7 ± 9.0 μg/L and 1.0% at 227.0 ± 15.0 μg/L. With no official criteria for iodine sufficiency in individuals, pregnant women were classified into an iodine-deficient group (UIC < 150 μg/L) and iodine-sufficient group (UIC ≥ 150 μg/L) based on WHO criteria [22]. With reference to the study by Bath et al. [28], which divides women with urinary iodine-to-creatinine ratio < 150 μg/g category into those with iodine concentrations < 50 μg/g and those with concentrations of 50–150 μg/g, the groups were further divided by UIC as < 100, 100–149, 150–249, and ≥ 250 µg/L.

### Statistical Analysis

Variables (FT3 and FT4) that were normally distributed data were expressed as mean ± standard deviation. Median (P25, P75) was used to report non-normally distributed data (UIC, Tg, and TSH). Percentages were used to express categorical variables. When comparing three or more groups of normally distributed data, analysis of variance was used, followed by the Bonferroni correction for multiple comparisons. The Kruskal–Wallis *H* test was used to compare non-normally distributed data when three or more groups were involved, followed by the Dunn-Bonferroni test for comparisons between two groups. Spearman’s rank correlation was used for analyzing correlations.

In order to explore whether the association of gestation week and TSH and Tg would vary with the iodine concentrations of pregnant women, linear mixed effects models were used to analyze the associations of iodine concentrations and gestation week on both TSH and Tg. In this model, TSH or Tg as the dependent variables, iodine concentration groups as fixed effect, and gestational week as random effect for gestational age differ in pregnant women while controlling for maternal age, body mass index (BMI), highest educational level, and passive smoking cigarette. Log-transformed TSH and Tg were used for analysis.

To test the robustness of the model, sensitivity analysis was conducted by further dividing maternal urinary iodine concentrations into four groups (< 100, 100–149, 150–249, and ≥ 250 µg/L) to analyze the effects of iodine concentrations (four groups) and gestational age on both TSH and Tg. We calculated the geometric mean ratios of TSH and Tg after controlling for multiple variables. The IBM SPSS statistical version 25.0 (IBM Corp., Armonk, NY, USA) was used to conduct the statistical analysis. A difference was considered statistically significant if *p* < 0.05.

## Results

### Participant Characteristics

The demographic characteristics of 1332 study participants and the correlation between the characteristics and Tg are outlined in Table 1. The mean age and gestational week at enrollment were 28.75 ± 4.35 years and 8.80 ± 1.41 weeks, respectively. 98.9% were Han ethnicity. 57.8% of the study population has a bachelor’s degree or above. Pregnant women aged < 35 years had a higher median serum Tg than those aged ≥ 35 years (*p* < 0.001); women with BMI < 18.5 kg/m^2^ had higher serum Tg than those with BMI 18.5–24.0 kg/m^2^ (*p* = 0.022); serum Tg was higher in pregnant women with ≥ 13 years of highest education than in pregnant women with ≤ 9 years of highest education (*p* = 0.010). There was no significant difference in serum Tg concentrations between pregnant women who were passive smokers and those who were not. The mean gestational week of samples was 9.63 ± 1.02 weeks, 17.12 ± 0.99 weeks, and 32.03 ± 0.31 weeks, respectively. The median UIC at each trimester was 116.08 (75.53, 171.60) μg/L, 111.20 (74.15, 163.51) μg/L, and 105.76 (68.93, 157.85) μg/L respectively. The median Tg concentration was 11.56 (7.39, 17.83) μg/L, 11.45 (7.37, 17.99) μg/L, and 12.43 (7.48, 19.22) μg/L in the first, second, and third trimesters, respectively. TSH and Tg increased and UIC, FT3, and FT4 decreased throughout the pregnancy (Table 2).

**Table 1:** The associations between the characteristics of 1332 study participants and Tg

Characteristics		*N* (%)	Tg (μg/L)	
			Median (P25, P75)	*p* value
Gestational age at enrollment (weeks)^a^	8.80 ± 1.41	1332 (100.00)	11.56 (7.39, 17.83)	-
Age at enrollment (years)	< 35	1196 (89.80)	11.72 (7.78, 18.14)	< 0.001
	≥ 35	136 (10.20)	9.68 (5.42, 14.85)	
BMI (kg/m^2^)	< 18.5	158 (11.80)	12.33 (9.00, 19.13)^b^	0.022
	18.5–24.0	912 (68.50)	11.35 (7.19, 17.48)^c^	
	≥ 24.0	262 (19.70)	11.22 (7.17, 18.16)^b,c^	
Ethnic groups	Han	1317 (98.90)	11.61 (7.39, 17.86)	0.050
	Others	15 (1.10)	8.22 (4.98, 13.04)	
Highest educational level (years)	≤ 9	211 (15.80)	10.15 (6.34, 16.17)^b^	0.010
	10–12	352 (26.40)	11.09 (7.35, 17.26)^b,c^	
	≥ 13	769 (57.80)	11.92 (7.89, 18.51)^c^	
Income (USD)^	< 9000	166 (12.50)	11.27 (8.08, 17.92)	0.983
	9000–14,999	234 (17.50)	12.25 (7.21, 17.78)	
	≥ 15,000	932 (70.00)	11.48 (7.38, 17.83)	
Passive smoking cigarette*	No	1063 (79.60)	11.67 (7.39, 18.07)	0.426
	Yes	272 (20.40)	11.01 (7.30, 17.37)	

^a^Values are mean ± SD; *the question was only asked at enrollment

^Household net income per capita in 2018

^b,c^Statistically significant differences for data in the same row with different superscript letters

**Table 2:** Urinary iodine concentrations and thyroid function by pregnancy

Variables	First trimester	Second trimester	Third trimester	*p* value
*N*	1332	1182	1026	-
UIC (μg/L)^a^	116.08 (75.53, 171.60)^c^	111.20 (74.15, 163.51)	105.76 (68.93, 157.85)^d^	0.003
Tg (μg/L)^a^	11.56 (7.39, 17.83)	11.45 (7.37, 17.99)	12.43 (7.48, 19.22)	0.033
TSH (mIU/L)^a^	1.15 (0.64, 1.73)^c^	1.71 (1.21, 2.32)^d^	1.81 (1.37, 2.49)^e^	< 0.001
FT3 (pmol/L)^b^	4.86 ± 0.59^c^	4.39 ± 0.56^d^	3.87 ± 0.53^e^	< 0.001
FT4 (pmol/L)^b^	16.98 ± 2.22^c^	14.13 ± 1.94^d^	12.26 ± 1.77^e^	< 0.001

^a^Values are median (P25, P75); ^b^values are mean ± SD

^c,d,e^Statistically significant differences for data in the same row with different superscript letters; *p* < 0.05 from the analysis of variance (ANOVA) or Kruskal–Wallis *H* test for continuous data

### Association of Iodine Status with Serum Tg and TSH

The correlations between UIC and Tg (*r* =  − 0.057, *p* = 0.001) and FT4 (*r* = 0.059, *p* < 0.001) were statistically significant but relatively weak throughout pregnancy. None of the correlations reached significance in the first trimester and second trimester. During the third trimester, UIC was inversely associated with Tg (*r* =  − 0.129, *p* < 0.001).

The linear mixed effects models were constructed to explore the changing trends in TSH and Tg between UIC groups with increasing gestational weeks. TSH and Tg were not statistically different in the iodine-deficient group compared to the iodine-sufficient group in terms of main effects (Table 3). Then, we analyzed the interaction between the iodine concentrations groups and gestation week. In both the TSH and Tg models, the interaction analysis showed that the effects between the iodine status groups and gestation week were significant (*p* = 0.038 and *p* = 0.007, respectively). TSH increased with the advancing gestational week in both iodine level groups. Tg did not significantly increase over the course of pregnancy in the iodine-adequate group, whereas there was a substantially increase in the iodine-inadequate group (Table 4).

**Table 3:** TSH and Tg between iodine level groups in terms of main effects in the linear mixed effects models*

	UIC group		*p* value
	< 150 μg/L	≥ 150 μg/L	
*n*	2445	1095	-
TSH (mIU/L)	0.032 (− 0.009, 0.073)	Reference	0.130
Tg (μg/L)	− 0.010 (− 0.034, 0.014)	Reference	0.411

*TSH and Tg were log-transformed. Values are expressed as *β* (95% CI), adjusting for the maternal age, BMI, highest educational level, passive smoking cigarette, and interaction regarding the iodine status and gestation week

**Table 4:** The effect on TSH and Tg concentrations of the interaction between iodine status and gestational week*

	UIC group	*n*	TSH			Tg	
			*β* (95% CI)	*p* for interaction		*β* (95% CI)	*p* for interaction
One-week increase in gestation	< 150 μg/L	2445	0.010 (0.007, 0.013)	0.038		0.002 (0.001, 0.003)	0.007
	≥ 150 μg/L	1095	0.012 (0.010, 0.013)			0.000 (− 0.001, 0.001)	

*TSH and Tg were log-transformed, controlling for maternal age, BMI, highest educational level, and passive smoking cigarette

As the interaction regarding iodine concentrations and advancing gestation week had statistically significant for TSH and Tg, simple effects analysis was further performed. There was little variation in TSH concentration in both iodine status groups at three trimesters. By contrast, Tg was higher in the iodine-deficient group than in the iodine-sufficient group at each trimester, with the difference between the groups being greatest in the third trimester (Table 5).

**Table 5:** TSH and Tg between iodine level groups at different trimesters*

Variables	Trimester	UIC < 150 μg/L		UIC ≥ 150 μg/L		*p* value
		*n*	Estimated marginal mean (95% CI)	*n*	Estimated marginal mean (95% CI)	
TSH (mIU/L)	First trimester	887	0.99 (0.94, 1.04)	445	1.01 (0.95, 1.08)	0.514
	Second trimester	815	1.63 (1.55, 1.71)	367	1.60 (1.49, 1.72)	0.742
	Third trimester	753	1.75 (1.66, 1.84)	283	1.86 (1.72, 2.02)	0.181
Tg (μg/L)	First trimester	887	10.38 (9.77, 11.02)	445	9.91 (9.14, 10.74)	0.347
	Second trimester	815	10.47 (9.86, 11.14)	367	9.98 (9.12, 10.89)	0.336
	Third trimester	743	11.91 (11.17, 12.68)	283	9.82 (8.89, 10.84)	0.001

*TSH and Tg were log-transformed. Values are estimated marginal mean (95% CI). Results are computed by back transformation of estimated marginal means, controlling for maternal age, BMI, highest educational level, and passive smoking cigarette

In sensitivity analysis, there were significant interactions between iodine status (four groups) and increasing gestation week on both TSH (*p* = 0.013) and Tg (*p* = 0.023). In four iodine status groups, TSH improved as the gestational weeks advanced. Tg increased with advancing pregnancy both in the < 100 μg/L group and 100–149 μg/L group, whereas not significantly in the 150–249 μg/L group and ≥ 250 μg/L group. The increase was greater in the < 100 μg/L group than in the 100–149 μg/L group (Table 6).

**Table 6:** The effect on TSH and Tg concentrations of the interaction between iodine status (in four groups) and gestational week*

	UIC group	*n*	TSH			Tg	
			*β* (95% CI)	*p* for interaction		*β* (95% CI)	*p* for interaction
One-week increase in gestation	< 100 μg/L	1511	0.009 (0.008, 0.010)	0.013		0.002 (0.002, 0.003)	0.023
	100–149 μg/L	934	0.012 (0.010, 0.013)			0.001 (0.000, 0.002)	
	150–249 μg/L	735	0.012 (0.010, 0.014)			0.000 (− 0.001, 0.002)	
	≥ 250 μg/L	360	0.011 (0.009, 0.014)			0.001 (− 0.001, 0.002)	

*TSH and Tg were log-transformed, controlling for maternal age, BMI, highest educational level, and passive smoking cigarette

## Discussion

The aim of this study was to explore the usefulness of maternal serum Tg as a biomarker of iodine status in pregnancy with mild-to-moderate iodine deficiency based on a repeated-measure study design. According to the criteria of the WHO, the median UIC values of < 150 μg/L at all trimesters suggested that the pregnant women were regarded as iodine deficient [22].

This result was in accordance with that from previous studies among pregnant women in Zhejiang Province, China [29]. Median UIC values of < 150 μg/L were similar to previous studies in pregnant women of Shanghai, China [30], including one Pakistan study which reported the UIC concentrations of the pregnant women remained mild iodine deficiency [31]. In our study, the median Tg concentrations were 11.56, 11.45, and 12.43 μg/L in the first, second, and third trimesters, respectively. Similar to the studies of Dineva et al., the Tg concentrations in population with mild-to-moderate iodine deficiency were 11.5 μg/L [32]. In the Northern Ireland study, the median trimester–specific Tg concentrations were 19 µg/L, 16 µg/L, and 16 µg/L, respectively [33], all of which were higher than the values of Tg in our study, which may be due to the fact that the overall UIC of pregnant women in this study was lower than that in our study. In addition, it has also been shown that the Tg concentrations of the pregnant women with UIC 150–249 μg/L were about 10.18 μg/L [20]. A study investigated the serum Tg in pregnancy as an iodine status biomarker in settings of iodine sufficiency and mild-to-moderate deficiency, where the median serum Tg of pregnant women in the groups with sufficient iodine intake was around 10 μg/L [32]. Moreover, Nazeri and colleagues [24] systematically reviewed the literature on urinary iodine and Tg in pregnant women; its results have shown that Tg concentration in iodine-deficient pregnant women (10.73 µg/L [5.65, 15.82]) was higher than that in iodine-sufficient pregnant women (7.34 µg/L [2.20, 12.47]). This suggests that pregnant women with suboptimal iodine intake have significantly higher Tg concentrations compared to those with adequate iodine intake and Tg has potential as an indicator for assessing iodine nutrition of pregnant women.

We found that the influence of increasing gestational weeks on TSH and Tg diverges in both iodine status groups. TSH increased with the gestational week in all iodine status groups, and TSH did not significantly differ between the two groups. These results were consistent with the findings from a recent randomized controlled trial (RCT) [34]. TSH concentrations increased for pregnancy in all groups, which can be explained by the well-known physiological change. TSH release is suppressed by human chorionic gonadotropin (hCG) in early pregnancy, and then, TSH increases in the second and third trimesters [35]. Besides, there was a considerable lag effect for TSH concentrations due to the regulation of thyroid hormones by the hypothalamus–pituitary–thyroid axis during mild iodine deficiency [36]. These reflect the limitation of TSH as a biomarker of iodine status in cases of mild-to-moderate iodine deficiency in pregnant women. Inconsistent with changes in TSH, Tg did not increase with advancing gestation in the ≥ 150 μg/L group but did in the < 150 μg/L group. And Tg concentrations were higher in the iodine-deficient group than in the iodine-sufficient group, with the difference between the groups being greatest in the third trimester. Several studies have shown that there was a stimulatory effect on thyroid function due to low iodine to maintain normal thyroid function [37]. This means that with the advance of pregnancy, if iodine intake fails to remain sufficient, the stores of iodine will be rapidly depleted contributing to a significant increase in serum Tg concentrations in the latter stages of pregnancy [38]. On the other hand, Tg concentrations during pregnancy might depend on the quantity of iodine stored in the thyroid gland and the initial iodine level [32]. However, it should be highlighted that Tg is not a specific indicator of iodine deficiency; even if the iodine intake was sufficient, its concentration might be influenced by thyroid secretory activity and the peak in hCG at the end of the first trimester [39]. Taken together, serum Tg may be used as a sensitive biomarker and a better indicator of iodine nutrition than TSH for pregnant women with suboptimal iodine status.

Tg is synthesized by thyroid cells and plays a crucial role in the generation of thyroid hormones such as T3 and T4 [40]; only minute amounts of Tg are released from a healthy thyroid [41]. When the body is suffering from iodine deficiency, low concentrations of circulating T4 stimulate the hypothalamic pituitary gland to release thyrotropin, increasing the production of TSH and an increase in serum Tg concentration. In this situation, iodine nutrition over a period of months or years can be reflected by the serum Tg concentration [22]. In addition, Tg is considered to be a biomarker of iodine deficiency in children and adults since it correlates well with UIC and thyroid volume [42, 43]. Other studies have also shown that Tg could be a biomarker of pregnant women with suboptimal iodine status [44]. In a longitudinal study, there was little increase in Tg during gestation in iodine-sufficient areas, whereas Tg increased and remained at a higher level with advancing pregnancy in iodine-deficient areas [45]. Overall, these studies and our study suggested that Tg shows promise as a sensitive indicator of iodine status in pregnant women with iodine deficiency and would be complementary to UIC measurement.

This study has the following limitations. Firstly, cluster sampling was used to select the study population in this study, and there might be selection bias. In addition, although three repeat spot UIC measures were available throughout pregnancy, these were not combined to provide an overall iodine status measure in pregnancy and iodine status at each trimester was based on a single-spot urine UIC measure. Single-spot UIC was highly variable and difficult to represent the iodine nutrition of individual pregnant women. Thirdly, Tg reflects the iodine status in weeks–months prior to the measurement, whereas spot UIC reflects the iodine status in preceding 24–48 h [6], so comparing Tg in early pregnancy with spot UIC in early pregnancy does not reflect iodine intake in the same time period. And it might be difficult to instantly reflect individual iodine status using Tg concentrations. However, the variation of serum Tg concentrations among individuals was lower than that of urinary iodine [16], suggesting that Tg might be a promising indicator of individual iodine nutrition assessment. Finally, thyroid hormones are disturbed by hCG in the first trimester, and we did not measure hCG concentrations during pregnancy, so we could not analyze hCG concerning iodine status and thyroid tests.

## Conclusions

In conclusion, we demonstrated that for pregnant women with mild-to-moderate iodine deficiency, Tg may be a more sensitive biomarker of iodine status than TSH. Further studies with more detailed information including hCG concentrations at each trimester are warranted to validate these findings and a multi-indicator system combined with serum iodine, urinary iodine, serum Tg, and iodine intake for a more accurate and precise assessment of iodine nutrition in pregnant women.

## Data Availability

The data presented in this study are available on request from the corresponding author. The data are not publicly available due to it involves personal information.
